# Suture augmentation of acromioclavicular and coracoclavicular ligament reconstruction for acute acromioclavicular dislocation

**DOI:** 10.1097/MD.0000000000027007

**Published:** 2021-08-20

**Authors:** Yingliang Liu, Xu Zhang, Yadong Yu, Weifeng Ding, Yong Gao, Yanting Wang, Rong Yang, Vikas Dhawan

**Affiliations:** a Department of Orthopaedics, People's Hospital of Chuxiong Yi Autonomous Prefecture, Yunnan, China; b Department of Hand Surgery, Third Hospital of Hebei Medical University, Shijiazhuang, Hebei, China; c Clinical Medicine Department of Dali University, Yunnan, China; d Hand and Microsurgery, Department of Orthopaedic Surgery, Saint Louis University School of Medicine, SLU Academic Pavilion 1008 S. Spring Avenue, St. Louis, MO.

**Keywords:** acromioclavicular ligament, coracoclavicular ligament, dislocation, Rockwood

## Abstract

The objective of this report was to introduce a new suture augmentation of coracoclavicular (CC) and acromioclavicular ligament reconstruction for acute Rockwood grade III to V acromioclavicular dislocations.

From January 2015 to January 2019, 43 patients with Rockwood III to VI acute acromioclavicular dislocations were retrospectively reviewed. For comparison, another series of 28 patients treated with double Endobutton technique from January 2011 to December 2014 were reviewed. A *P* < .05 was considered statistical significance.

The mean follow-up period of the 2 series were 39.69 ± 7.42 months (range, 24–54 months) and 37.86 ± 8.23 months (range, 26–48 months) (*P* > .05), respectively. There were significant differences regarding CC space (11.62 ± 2.54 mm vs 16.78 ± 5.53 mm; *P* < .05), CC reduction loss (5.56 ± 4.73 mm vs 26.25 ± 4.42 mm; *P* < .05), and acromioclavicular space (6.89 ± 1.87 mm vs 7.95 ± 2.37 mm; *P* < .05). There were significant differences regarding the disabilities of the arm, shoulder, and hand questionnaire (3.3 ± 2.8 vs 5.32 ± 4.37; *P* < .05) and University of California–Los Angeles shoulder rating scale (31.19 ± 2.48 vs 29.24 ± 2.48; *P* < .05). The excellent to good percentages were 100% (n = 32) and 85% (n = 23), respectively.

In conclusion, the suture augmentation of acromioclavicular and CC ligament reconstruction is a reliable technique for acute acromioclavicular dislocation with minimal complications.

Type of study/level of evidence: Therapeutic IIa.

## 1 Introduction

Acute acromioclavicular (AC) dislocation typically occurs in young, athletic adults and is one of the most common injuries of the shoulder girdle (4%–12%).^[1]^ The ideal surgical techniques for treating high-grade acute AC injuries are a matter of ongoing debate.^[2]^

The original classification of AC injuries was described by Rockwood and Green according to the injured ligament complex, as well as the degree and direction of clavicular displacement.^[3]^ Low-grade sprains (type I and II) are usually managed nonoperatively.^[4]^ Patients with type III injuries are usually evaluated on a case-by-case basis, taking into account the variables like hand dominance, age, occupation, sport requirements, dysfunction, and risk of redislocation.^[3]^ More high-grade AC instability (type IV and VI) are frequently the result of high-energy injuries. The injuries are complex and may cause persistent shoulder pain and functional impairment.

Many surgical procedures have been described for treating AC dislocations. Among those are screws, plates, muscle transfer, ligamentoplasty procedures, and ligament reconstruction using autografts or allografts.^[5]^ In addition, with the advancement of shoulder arthroscopy, surgeons are much more capable of performing mini-open or arthroscopically-assisted procedures, allowing patients an earlier return to their daily living activities.^[6]^ However, the results of conventional open techniques are still comparable. Currently, there is no gold standard for the surgical treatment of AC injuries because comparison among the techniques is difficult due to limited patients.

The objective of this report was to introduce a new suture augmentation of coracoclavicular (CC) and AC ligament reconstruction for acute Rockwood grade III to V AC dislocations. For comparison, we reviewed another series of 28 patients treated with the double Endobutton technique because it is commonly used for treating AC dislocation.

## 2 Materials and methods

This retrospective study was approved by the institutional review boards of the hospitals involved in accordance with international agreements (World Medical Association Declaration of Helsinki “Ethical Principles for Medical Research Involving Human Subjects,” amended in October 2013, www.wma.net). Informed consent and Health Insurance Portability and Accountability Act consent were obtained from each patient.

**Figure 1:**
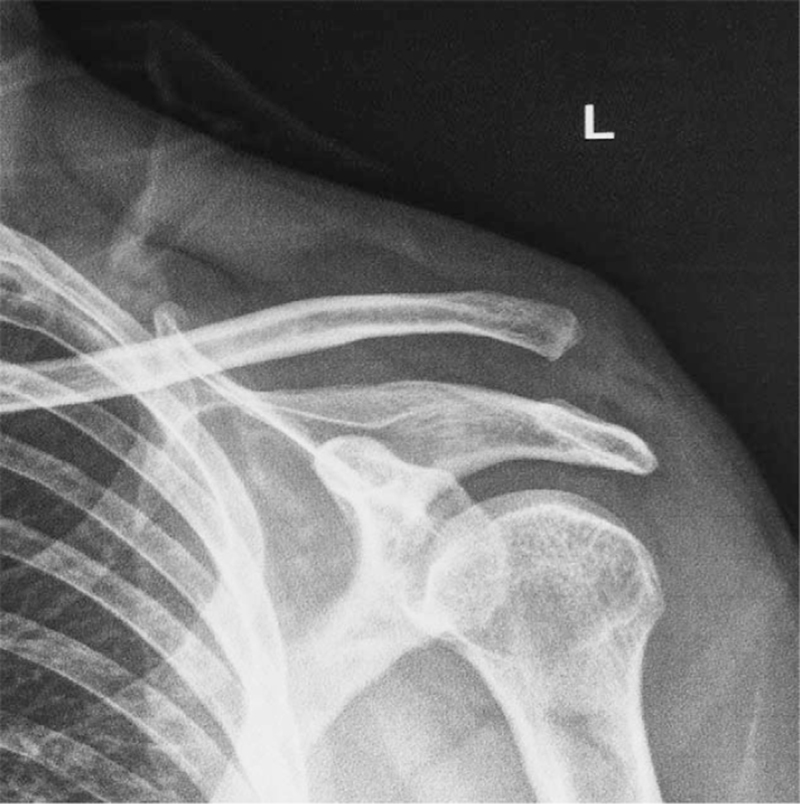
A 47-year-old male patient suffers an acute acromioclavicular (AC) dislocation (left side; anteroposterior view) in a road traffic accident.

From January 2015 to January 2019, 43 patients with Rockwood III to VI acute AC dislocations were retrospectively reviewed. The diagnosis was established according to the proper history taking, physical examination, and X-ray (Fig. 1). An increase of CC distance of 25% to 50% over the normal side on a bilateral Zanca view indicated complete CC ligament disruption.

Our eligibility criteria were: patients aged between 18 and 65 years; an acute AC dislocation within 14 days; Rockwood grade III to VI; and normal opposite upper limb for comparison. Our exclusion criteria were as follows: patients younger than 18 years were excluded because of skeletal immaturity (n = 3); patients older than 65 years are excluded because of possible osteoporosis (n = 1)^[7]^; combined fractures of the joints (n = 1); old AC dislocations exceeding 14 days because the treatments may be different; discontinued intervention (n = 4); and declined to participate (n = 2). Finally, a total of 32 patients were enrolled in this study. All operations were performed by the same senior orthopaedic surgeon.

### 2.1 Surgical technique

**Figure 2:**
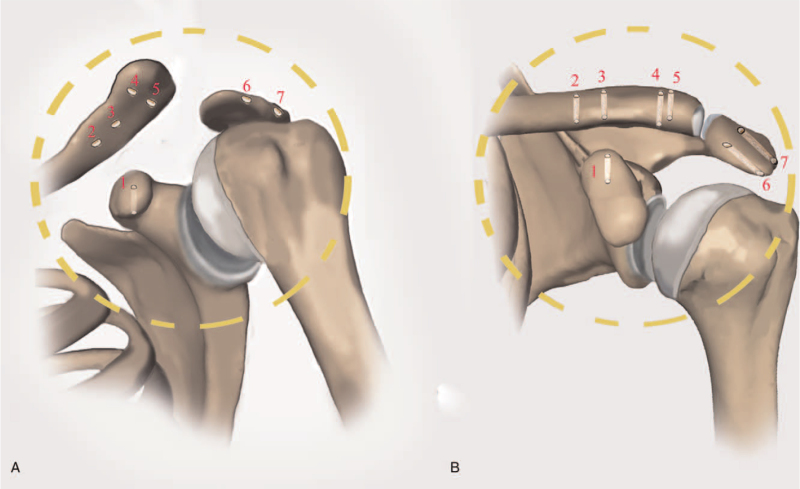
A total of 7 bicortical tunnels (numbered from #1–#7) are made (the images in the dashed circle will be enlarged in the next figure). A. A 15° cephalic Zanca view showing the left AC dislocation. B. An anterior view showing the clavicle is reduced.

**Figure 3:**
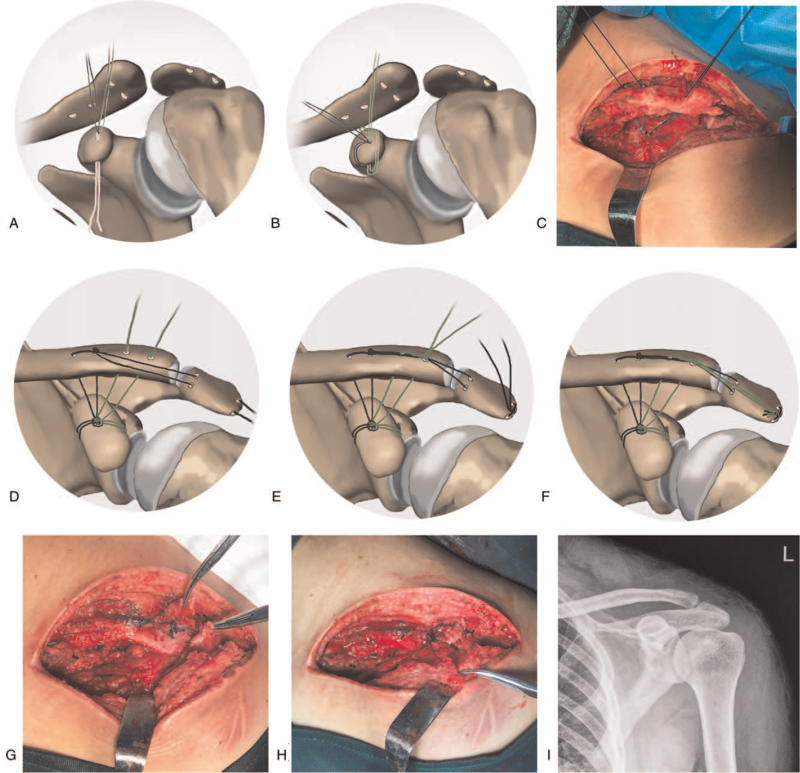
The systematical ligament reconstruction. A. A cephalic Zanca view showing 2 2/0 polyester sutures (marked black and green separately for better understanding) passed through the coracoid tunnel (#1) using a 1 mm stainless steel wire loop (white wire). B. Each suture is passed through the loop of the same suture and then tightened by pulling the free limbs. The 4 limbs are tied together. C. The 4 limbs are passed through the 4 clavicle tunnels separately. D. An anterior view showing the 2 black limbs passed through the proximal clavicle tunnels (#2 and #3) are tied to each other over the clavicle and further passed through the 2 acromion tunnels (# 6 and #7). E. The 2 green limbs passed through the distal clavicle tunnels (#4 and #5) are tied to each other over the black limbs to prevent them slide anteriorly. F. The 2 black limbs are tied over the lateral acromion, and then the 4 limbs are tied together over the lateral acromion. G. The AC and CC ligaments are repaired. H. Ligament reconstruction and repair are complete. I. Anteroposterior X-ray immediately after surgery. AC = Acute acromioclavicular, CC = coracoclavicular.

The operation was performed with the patient under general anesthesia. The patient was placed in the beach chair position or supine position with extra padding under the injured shoulder. An 8-cm curved incision was made from the AC joint to the distal anterior clavicle. The base of the coracoid process, AC joint, and distal clavicle were visualized. One coracoid tunnel (#1), 4 clavicle tunnels (#2–#5), and 2 acromion tunnels (# 6 and #7) were drilled using a 2.0 mm pin (Fig. 2A). The lateral clavicle was reduced by manipulation (Fig. 2B), and maintained using pointed reduction clamps or a K-wire as needed. We passed 2 2/0 braided nonabsorbable polyester sutures (Ethicon, INC., Somerville, NJ, USA) through the coracoid tunnel (#1) using a 1 mm stainless steel wire loop (Fig. 3A). The 2 free limbs of each suture were passed through the loop of the same suture and then tightened over the coracoid (Fig. 3B). The 4 limbs were tied together and then passed through the clavicle tunnels (#2–#5) separately to reconstruct the CC ligament (Fig. 3C). Two of the limbs were tied to the corresponding pair (Fig. 3D, E). Two limbs passed through the acromion tunnels (# 6 and #7), and another 2 limbs were tied together over the acromion to reconstruct the AC ligament (Fig. 3F). The ruptured AC, CC, and coracoid ligaments, as well as the capsule, were repaired if possible (Fig. 3G, H). The temporary maintenance was removed, and the AC reduction was confirmed on X-ray (Fig. 3I). The wound was closed in layers.

### 2.2 Postoperative management

After surgery, the limb was supported with a platform brace for 6 weeks to minimize the gravity-induced stress on the AC joint. Gentle passive range of motion was started after 6 weeks. Strength exercises for the scapular muscle were started after 12 weeks.

### 2.3 Outcome evaluation

Active motions of the shoulder were measured with a goniometer, and all measurements were compared to those on the opposite limb. On the frontal X-ray of the shoulder, the CC space (distance between the superior cortex of the coracoid process and the undersurface of the clavicle) was assessed.^[8]^ Loss of reduction was defined as >25% increase of CC distance developed. The AC space was also assessed. The grip strength of the hand was measured using a dynamometer. To improve consistency between dominant and nondominant grip strength, we based the scores for analysis on the premise that the grip strength was 6% higher at dominant sides compared with the nondominant sides.^[9]^ We used the disabilities of the arm, shoulder, and hand questionnaire^[10]^ to assess limb function. Clinical evaluation of patients was performed using the University of California–Los Angeles (UCLA)^[11]^ scoring systems. The UCLA score consisted of pain (0–10 points), function (0–10 points), range of motion (0–5 points), strength (0–5 points), and the patient's satisfaction (0–5 points). The total UCLA score is 35 points, and 34 points or 35 points, 29 points to 33 points, and ≤29 points indicate excellent, good, and poor results, respectively.

### 2.4 Statistical analysis

Quantitative variables were described as mean and ranges. The collected data were analyzed with the statistical package for social sciences 19.0 (SPSS, Inc., Chicago, Ill). A *P* < .05 was considered statistical significance.

## 3 Results

**Figure 4:**
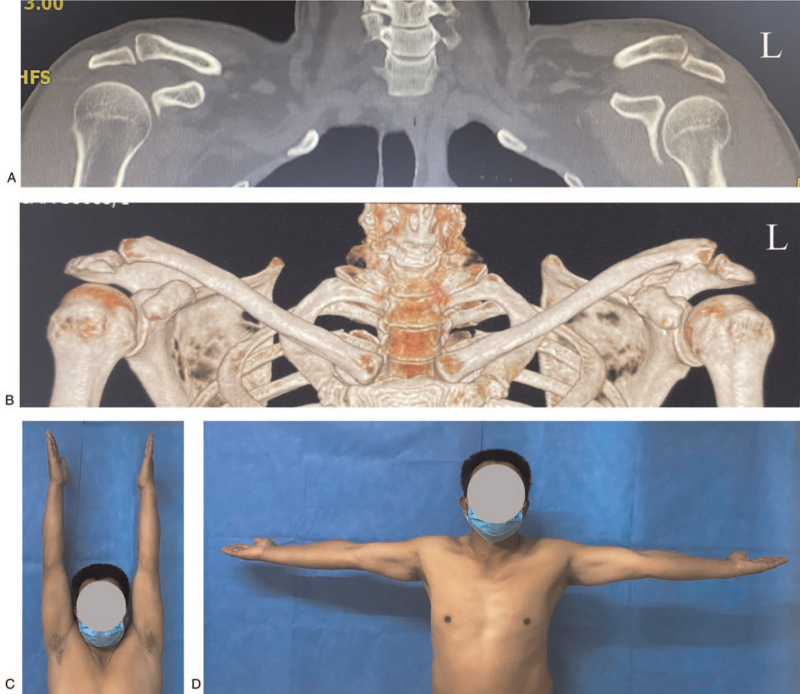
Two years after surgery. A. Coronal CT. B. Three-dimensional CT. C. Vertical flexion. D. Abduction. CT = computed tomography.

There were 28 male and 4 female patients who underwent multiple ligament reconstruction and repair. These patients were comprised of group A. The mean age at surgery was 34 years (range, 18–54 years). The causes of injuries included high-energy contact sports (n = 22), road traffic accident (n = 8), and fall from a height (n = 2). The AC injuries included type III (n = 2), IV (11), V (n = 19), and VI (0) dislocations. (Table 1) No patient was lost to follow-up, and all 32 patients were reviewed with an average follow-up of 39.69 ± 7.42 months (range, 24–54 months) (Fig. 4A–D).

**Table 1:** The baseline data of 2 groups.

	Group A (n = 32)	Group B (n = 28)	
Techniques	MLRR	Double Endobutton	*P* value
Age (yr, mean, range)	34 (18–54)	32 (18–57)	.155
Sex (m: f)	28: 4	26: 2	.205
Injured side (R: L)	17: 15	13: 15	.295
Dominance (n)	19: 13	18: 10	.05
TFITS (d; mean, range)	6 (3–10)	7 (4–13)	.132
Causes (n)			
Sports	22	17	.625
Traffic accident	8	6	
Fall from a height	2	5	
Rockwood classification (n)			
III	2	1	.423
IV	11	7	
V	19	20	
VI	0	0	

MLRR = multiple ligament reconstruction and repair, TFITS = time from injury to surgery.

For comparison, we retrospectively reviewed 28 patients who underwent AC ligament reconstruction and repair using the double Endobutton technique from January 2011 to December 2014. These patients were comprised of group B. The mean age at surgery was 32 years (range, 18–57 years). The patients were operated within 14 days of injury. The causes of injuries included high-energy contact sports (n = 17), road traffic accident (n = 6), and fall from a height (n = 5). The AC injuries included type III (n = 1), IV (7), V (n = 20), and VI (0) dislocations. No patient was lost to follow-up, and all 28 patients were reviewed with an average follow-up of 37.86 ± 8.23 months (range, 26–48 months).

There were no significant differences with regard to patient age, sex, cause of injury, type of injury, preoperative CC space, and duration of follow-up. We found significant differences regarding vertical flexion (93.22 ± 6.68 vs 94.52 ± 4.25; *P* = .006), external rotation (92.35 ± 7.62 vs 79.53 ± 23.02; *P* = .000), and internal rotation (90.42 ± 10.55 vs 87.22 ± 6.36; *P* = .000) of the shoulder. We found significant differences regarding CC space (11.62 ± 2.54 mm vs 16.78 ± 5.53 mm; *P* = .000), CC reduction loss (5.56 ± 4.73 mm vs 26.25 ± 4.42 mm; *P* = .000), and AC space (6.89 ± 1.87 mm vs 7.95 ± 2.37 mm; *P* = .000) at the final follow-up. We also found significant differences regarding disabilities of the arm, shoulder, and hand questionnaire (3.3 ± 2.8 vs 5.32 ± 4.37; *P* = .012) and UCLA shoulder rating scale (31.19 ± 2.48 vs 29.24 ± 2.48; *P* = .000). The excellent to good percentages were 100% (n = 32) and 85% (n = 23), respectively (Table 2).

**Table 2:** Outcomes at the final follow-up.

	Group A	Group B	
	(n = 32)	(n = 28)	*P* value
Follow-up (mo; mean ± SD; range)	39.69 ± 7.42 (24–54)	37.86 ± 8.23 (26–48)	.344
ROM (mean ± SD; %)^a^			
Abduction	91.45 ± 8.22	88.11 ± 11.65	.257
Vertical flexion	93.22 ± 6.68	94.52 ± 4.25	.006
Flexion	91.73 ± 8.11	90.38 ± 5.69	.071
Forward flexion	88.32 ± 12.56	85.84 ± 12.23	.053
External rotation	92.35 ± 7.62	79.53 ± 23.02	.000
Internal rotation	90.42 ± 10.55	87.22 ± 6.36	.000
CC space (mm)			
Preop	19.35 ± 3.37	18.85 ± 4.01	.133
Immediate postop	10.55 ± 1.62	13.78 ± 1.37	.054
Final follow-up	11.62 ± 2.54	16.78 ± 5.53	.000
CC reduction loss (mm)			
Immediate postop	4.11 ± 3.83	16.05 ± 9.23	.000
Final follow-up	5.56 ± 4.73	26.25 ± 4.42	.000
AC space (mm)			
Immediate postop	6.32 ± 1.93	7.28 ± 2.44	.000
Final follow-up	6.89 ± 1.87	7.95 ± 2.37	.000
Grip strength (%)^a^	98.22 ± 9.251	97.85 ± 12.57	.178
DASH	3.3 ± 2.8	5.32 ± 4.37	.012
UCLA			
Pain (0–10)	9.25 ± 0.82	7.56 ± 2.17	.000
Function (0–10)	9.02 ± 0.97	8.18 ± 1.59	.000
ROM (0–5)	4.25 ± 0.61	4.03 ± 1.37	.021
Strength (0–5)	4.62 ± 0.39	4.28 ± 1.43	.018
Satisfaction (0–5)	4.25 ± 0.69	3.17 ± 1.27	.000
Total (n; %)	31.19 ± 2.48	29.24 ± 2.48	.000
Excellent (34–35)	28 (88)	15 (54)	
Good (29–33)	4 (12)	8 (31)	
Poor (≤29)	0	5 (45)	

AC = acromioclavicular, CC = coracoclavicular, DASH = disabilities of the arm, shoulder and hand questionnaire, ROM = range of motion, UCLA = University of California–Los Angeles shoulder rating scale.

a Comparing to the opposite limb.

## 4 Discussion

The common mechanism of AC joint separation involves a direct trauma to the posterosuperior part of the shoulder or an indirect mechanism via a fall on an outstretched adducted arm or elbow, which drives the humeral head into the AC joint.^[2]^ This injury occurs 5 times more frequently in men than in women, with the highest incidence in the 20- to 30-year-old age group. The complete AC dislocations often involve disruption of the AC and CC ligaments. Patients usually complain of pain and tenderness over the shoulder, particularly over the AC joint.^[1]^ Stabilizing the AC joint is a challenging technique even several methods are reported in the literature. Currently, the controversy still persists on the optimal treatment because there is no gold standard for the treatment of AC dislocation.

The CC distance is an indicator of the integrity of the CC ligament.^[2]^ The CC space was assessed on the frontal X-ray of the shoulder or clavicle or the coronal projection of a CT or MRI as the distance between the superior cortex of the coracoid process and the undersurface of the clavicle where the CC ligaments insert. Currently, there is no gold standard for the surgical treatment of any type of AC injuries. In 1972, Weaver and Dunn described a surgical procedure for treating the unstable AC joint.^[12]^ The technique consists of excising the distal part of the clavicle, releasing the coracoacromial ligament from its acromial attachment, and transferring it to the distal clavicle. Although several modified techniques had been reported with good outcomes, the rates of loss of reduction were usually up to 20%.^[13]^ Anatomical reconstruction of the CA ligament using free grafts provides better stability than other ligament transfer procedures.^[14]^ However, biomechanical studies have suggested that the CA ligament is a weak graft that has approximately 25% of the initial strength of the native CC ligaments and less than 50% of the appropriate stiffness.^[15]^ These nondynamic reconstructions may be failed because the success depends on the primary healing of the CC ligament.^[16–18]^ The early surgical techniques for AC joint fixation using K-wires and pins can hold the clavicle in a reduced position. However, owing to the high complication rates of implant migration and loss of reduction, surgeons tend to avoid using the techniques.^[19]^ Restoring AC joint function using a single or double Endobuttons can reduce the stress-rising effects of titanium buttons around the clavicle and the coracoid, through which to minimize the risk of failure by suture cut-out.^[20]^ Open reduction and internal fixation with hook plate is an effective treatment for AC dislocation, but hook plate had a significant impact on shoulder function. Chen et al^[21]^ treated 33 patients using the hook plate and found subacromial osteolysis occurred in 10 patients and co-occurrence of subacromial osteolysis and acromioclavicular joint osteoarthritis in 4 patients. The introduction of new arthroscopic equipment provides various surgical procedures, though every new technique has its own advantages and pitfalls.^[6]^ Few reports have proposed arthroscopic techniques to treat AC dislocations. Different materials have been used to maintain the CC reduction, including suture loops, sutures and anchors, and various types of metallic pins, screws, or buttons. The surgeon's expertise is likely to be the most significant factor affecting the outcomes. Possible failures with recurrent clavicular subluxation or dislocation have been reported after some of these procedures, with failure rates up to 50%. Fixation failures have been found to be due to hardware breakage or migration, suture abrasion and breakage, or bone erosion because of the potential sawing action of the sutures through the clavicle or the coracoid.^[22]^

Some suturing techniques are also reported in the literature. Huang et al^[23]^ suspended suture augmentation through the drill holes in the clavicle, but they did not reconstruct the AC ligament. Cho et al^[24]^ used suture anchors to provide CC stability alone. Our technique reconstructed both the AC and CC ligaments, which is stronger than CC reconstruction alone. The sutures passing through the bone tunnels can hold the clavicle or coracoid more strongly than bone anchors. Although the suture looping around the coracoid process alone can hold the displaced clavicle, the suture passing the tunnel in the coracoid can effectively maintain the suture tension by avoiding it moving back and forth.

Each ligament is repaired with 4 strands of suture, which provides strong strength for maintaining the reduction. The suture method avoids a metal-to-bone impact that may cause postoperative shoulder pain. The strong suturing allows early joint motion exercises, resulting in a good shoulder function.

The advantages of our technique include strong sutures to support an early range of motion and rehabilitation, the possibility of early return to work, ligament healing and a nearly anatomic reconstruction, no hardware removal, and very low morbidity. The disadvantages included slightly complex surgical procedures. The technique cannot be complete through a minimally invasive approach.

The indications of our technique are almost the same as other surgical techniques for treating AC dislocation. Either acute or old Rockwood type IV and VI dislocations, as well as some type III dislocations, are good candidates. Either direct repairing the ligament or repairing with a graft can be used depending on the surgeon's preference. Contraindication is a combined fracture that requires a rigid fixation. In order to exclude the biases of the study, we excluded the complex wounds, through those injuries are not contraindication.

The limitation of the study is that kinematics of the sutures need further studies. Also, surgeon preference, experience, and ability may influence ascertaining the effects of the treatment.

In conclusion, the suture augmentation of AC and CC ligament reconstruction is a reliable technique for acute acromioclavicular dislocation with minimal complications.

## Author contributions

Conceived and designed the study: Yingliang Liu and Yadong Yu. Extracted the data: Yong Gao, MD., Yanting Wang, MD., and Rong Yang, MD., Vikas Dahwan. Contacted patients follow-up: Yanting Wang. Pictures: Rong Yang. Performed the statistical analysis: Yong Gao. Wrote the manuscript: Yingliang Liu. All authors read and approved the final manuscript.

**Conceptualization:** Xu Zhang, Yadong Yu.

**Data curation:** Yong Gao, Yanting Wang.

**Formal analysis:** Yong Gao.

**Investigation:** Xu Zhang, Weifeng Ding.

**Methodology:** Yingliang Liu, Yanting Wang.

**Project administration:** Vikas Dhawan.

**Resources:** Weifeng Ding, Yanting Wang.

**Supervision:** Yong Gao.

**Validation:** Xu Zhang, Rong Yang.

**Visualization:** Vikas Dhawan.

**Writing – original draft:** Rong Yang.

**Writing – review & editing:** Rong Yang.
